# Brain interstitial fluid pharmacokinetics and therapeutic effect of a BBB penetrating amyloid beta antibody measured by microdialysis

**DOI:** 10.1016/j.neurot.2026.e00949

**Published:** 2026-06-24

**Authors:** Elin Wik, Amelia D. Dahlén, Ulrika Julku, Mengfei Xiong, Wojciech Michno, Stina Syvänen, Dag Sehlin

**Affiliations:** a Department of Public Health and Caring Sciences, Uppsala University, Uppsala, Sweden; b Department of Department of Medicinal Chemistry, Uppsala University, Uppsala, Sweden

**Keywords:** Immunotherapy, Bispecific antibody, Amyloid-β, Transferrin receptor, Microdialysis, Blood–brain barrier

## Abstract

The disease-modifying antibody lecanemab for treating Alzheimer's disease (AD) was initially designed to target amyloid-beta (Aβ) protofibrils, i.e. soluble aggregates of Aβ, but it has also been successful in clearing insoluble amyloid plaques in clinical studies. Therefore, this study aimed to investigate how a brain penetrating, bispecific murine variant of lecanemab (RmAb158-scFv8D3) distributes in the brain and interacts with different pools of aggregated Aβ in APP transgenic mice. The alpha-synuclein targeting antibody RmAbSynO2-scFv8D3 was used as control. Further, by performing *in vivo* high cut-off microdialysis in freely moving animals, brain interstitial fluid (ISF) was continuously collected across 24 h to assess concentrations of free antibody in the brain. *Post mortem* distribution of the antibodies was analyzed by sequential extraction of brain tissue. RmAb158-scFv8D3 showed rapid ISF clearance as well as a redistribution from brain extracts containing small, soluble Aβ species toward brain extracts containing insoluble, plaque-associated Aβ with time. A treatment effect was detected already at 12 h post injection, whereby the RmAb158-scFv8D3-treated animals showed lower concentrations of the smallest, most soluble Aβ aggregates. Collectively, these findings suggest that within the first 24 h after a single injection of the bispecific RmAb158-scFv8D3 antibody we can capture the antibody's initial brain distribution and interactions with both soluble Aβ aggregates and insoluble, plaque-associated Aβ. These interactions mediate a swift reduction of soluble Aβ, while clearance of insoluble Aβ requires longer treatment time.

## Introduction

The monoclonal antibody lecanemab that is directed toward amyloid-beta (Aβ) was approved for treating Alzheimer's disease (AD) by the U.S. Food and Drug Administration (FDA) in 2023, making it the first globally available disease modifying drug against AD after decades of failed clinical trials. Lecanemab was initially designed to target soluble Aβ protofibrils and has approximately 200 times higher *in vitro* affinity to protofibrils over monomers and 10 times higher compared to fibrils [1]. The term ‘soluble Aβ protofibrils’ has in the context of lecanemab been defined as a large (>100 kDa) soluble Aβ assemblies that form as an intermediate during the aggregation of monomeric Aβ to insoluble fibrils [[2], [3], [4]]. These neurotoxic protofibrils are thought to play a crucial role in the disease and appeared as the main affected Aβ pool in the first preclinical therapy studies in a mouse model of Aβ pathology [5]. However, protofibrils or other soluble Aβ aggregates have never been reliably measured in samples from treated patients and their importance as therapeutic target therefore remains largely unknown. Instead, clinical trials indicate that reduced cognitive decline after lecanemab treatment is associated with a markedly reduced signal in amyloid positron emission tomography (PET) [6]. This suggests that lecanemab effectively engages with and clears insoluble Aβ plaques. Thus, it is of great interest to investigate how the antibody interacts with different Aβ species *in vivo* and how this interaction influences Aβ clearance. Further, the molecular and cellular mechanisms behind Aβ clearance following immunotherapy in AD remain partially unclear. Recent investigations report distinct microglial adaptations associated with antibody mediated Aβ clearance around parenchymal plaques in both human subjects and mouse models [[7], [8], [9]]. However, it is still unknown whether the same mechanism applies to antibody mediated clearance of soluble Aβ aggregates.

The approvals of lecanemab and donanemab constitute huge leap forward in the field of immunotherapy for neurodegenerative diseases, yet there are still several issues left to tackle. Firstly, Aβ antibody therapy has been associated with side-effects known as amyloid-related imaging abnormalities (ARIA) appearing as edema (ARIA-E) or hemorrhages (ARIA-H) that can be visualized with magnetic resonance imaging. ARIA is hypothesized to develop a consequence of antibody interactions with vascular Aβ deposits, which cause inflammatory reactions that may damage the blood-brain barrier and cause edema or hemorrhages. The occurrence of ARIA is associated with the mode of antibody entry into the brain. The large size of antibodies (150 kDa for immunoglobulin G (IgG)) greatly restricts their ability to cross the blood-brain barrier (BBB) [10]. As a result, only approximately 0.1% of the administered dose reaches the brain parenchyma, but is then confined to certain compartments, such as central periventricular areas or superficial leptomeningeal tissue [8,9,[11], [12], [13]]. This requires elevated antibody dosing, which in turn increases the incidence of ARIA and leads to high cost of antibody production [14].

An attractive approach to achieve effective antibody transport into the brain is transferrin receptor 1 (TfR) mediated transcytosis. By fusing a single chain fragment variable (scFv) of the TfR antibody 8D3 to an antibody, the resulting bispecific construct can engage with TfR at the BBB, facilitating a wide antibody distribution across the whole brain and interstitial fluid (ISF) [12,13,15,16]. This method greatly increases antibody delivery across the BBB throughout the whole brain capillary network, while at the same time reducing antibody interactions with perivascular Aβ deposits in larger vessels. This approach is expected to result in increased therapeutic efficacy even with a lower antibody dose, which in combination with a more favorable brain entry route may decrease the risk of ARIA [9]. When applied to RmAb158, the murine version of lecanemab, a 10-fold lower dose of the bispecific antibody achieved the same clearance of soluble Aβ aggregates as the unmodified RmAb158 [11]. Similarly, the bispecific antibody RmAbSynO2-scFv8D3, targeting aggregated α-synuclein, exhibited a broader brain distribution and reduction of α-synuclein than its monospecific variant RmAbSynO2 in the transgenic L61 mouse model of α-synuclein pathology [17,18]. To better understand the brain entry and distribution of these bispecific constructs in animal models of proteinopathies, microdialysis can be used to continuously measure the concentrations of free antibody in the ISF *in vivo*. The method relies on a semipermeable probe inserted into the brain region of interest. We have previously used microdialysis to compare the brain pharmacokinetics of the Aβ-targeting monospecific mAb3D6 and bispecific, brain-penetrating mAb3D6-scFv8D3 antibodies in the *App*^*NL-G-F*^ mouse model of Aβ pathology [15].

The current study aimed to investigate how RmAb158-scFv8D3, the bispecific murine version of lecanemab, distributes in the brain over the first 24 h after administration and how it interacts with and clears different pools of parenchymal Aβ aggregates. This was achieved through a combination of *in vivo* high cut-off microdialysis in freely moving animals and measurements of Aβ and antibody concentrations in *post mortem* brain tissue.

## Materials and Methods

### Antibodies

The antibodies, RmAb158-scFv8D3 [19] that targets Aβ and RmAbSynO2-scFv8D3 [18] that targets α-synuclein, were expressed as previously described [20]. Briefly, Expi293F mammalian cells were transiently transfected with pcDNA3.4 vectors carrying the sequences for either the heavy or light chains of the antibody variants. Polyethyleneimine (PEI) was used as the transfection reagent and valproic acid as the cell cycle inhibitor. The antibody was purified and separated from the cell medium with an ÄKTA system (GE Healthcare) and the buffer was subsequently exchanged to phosphate-buffered saline (PBS; pH 7.4).

### Radiochemistry

RmAb158-scFv8D3 and RmAbSynO2-scFv8D3 were radiolabeled with iodine-125 (^125^I) by the Chloramine T method [21]. In short, ^125^I stock solution (PerkinElmer Inc Waltham, MA, USA) was mixed with antibody, 5 μg Chloramine-T (Sigma Aldrich) and PBS to a final volume of 110 μL. The Chloramine T reaction was incubated for 90 s in room temperature (RT) before 10 μg sodium metabisulfite (Sigma Aldrich) was added to quench the reaction. The resulting product was separated from free iodine and low molecular weight products using Zeba spin desalting columns (7 K MWCO, 0.5 mL, 89882, Thermo Fisher).

### Animals

Male (n = 20) and female (n = 19) tg-ArcSwe mice (aged 11–18 months) were included in the study. The tg-ArcSwe model expresses human amyloid precursor protein (APP) with the Swedish (KM670/671NL) and Arctic (E693G) mutations on a C57BL/6 background. Tg-ArcSwe mice show increased levels of soluble Aβ protofibrils with onset of Aβ plaque pathology from 6 months of age [22,23]. Three animals were excluded throughout the study; one mouse had a too low probe recovery, one displayed bleeding in the brain and one had an incorrect probe placement. The mice were housed in animal facility at Uppsala University in individually ventilated cages with 12/12 h dark–light cycle and *ad libitum* access to food pellets and water. All animal experiments were approved by the Uppsala County Animal Ethics board (5.8.18-16493-2024) following the legislation and regulations of the Swedish Animal Welfare Agency and European Communities Council Directive of September 22, 2010 (2010/63/EU).

### Stereotaxic surgery

A guide cannula (CMA7 guide cannula, CMA Microdialysis AB, Kista Sweden) was inserted into the left hippocampus by stereotaxic surgery (−2.2 A/P, +1.2 L/M from bregma and −1.5 D/V from the dura). Dental cement (AgnTho's, Lidingö, Sweden) was applied to secure the guide cannula and to close the wound. The mice were anesthetized with isoflurane (induction 4% and maintenance 2%; Isoflurane Baxter, Baxter S.A., Lessines, Belgium) during the entire procedure. Meloxicam, 2 mg/kg (Metacam 2 mg/L, Boehringer Ingelheim, Stockholm Sweden), was administered subcutaneously as pre-operative systemic analgesia and 0.05–0.1 mL lidocaine (Xylocain 10 mg/mL, Aspen) was administered under the scalp for local pre-operative pain relief.

Buprenorphine 0.05–0.1 mg/kg (Bupaq 0.3 mg/mL Richter Pharma AG) was subcutaneously administered postoperatively before the mice were returned to their home cage. Further post-operative pain relief was administered up to 48 h after injection: Buprenorphine (0.1 mg/kg) 5–6 h after surgery and Meloxicam (2 mg/kg) 20–24 h and 48 h after surgery. The mice were allowed to recover from the surgery for one week before microdialysis. The placement of the guide cannula was visually verified in *post mortem* brain tissue after completion of the microdialysis experiment.

### Sampling of brain interstitial fluid by microdialysis

**Fig. 1:**
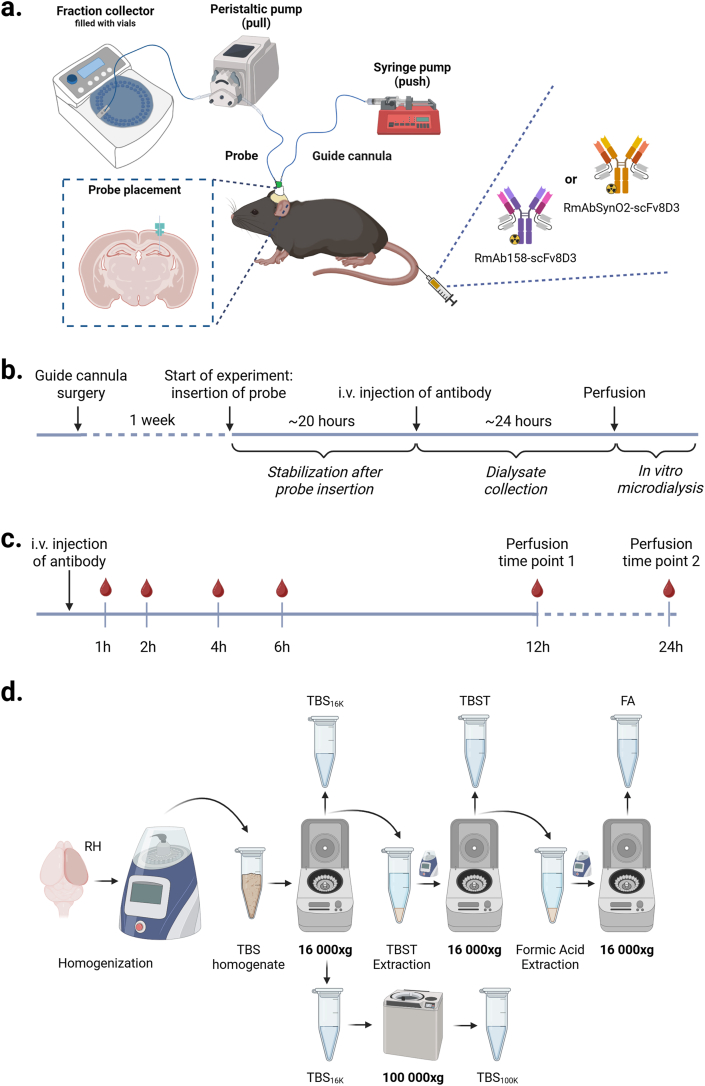
a. Intravenously injected bispecific [^125^I]RmAb158-scFv8D3 and [^125^I]RmAbSynO2-scFv8D3 were measured in the hippocampal interstitial fluid (ISF) using push-and-pull microdialysis. b. Timeline for the microdialysis experiments and c. Timeline for blood sampling post injection. d. The right cerebrum, contralateral to the implanted microdialysis probe, was homogenized and sequentially extracted to release soluble, membrane associated and plaque associated Aβ. Fig. 1 was created using BioRender

On the first day of the microdialysis experiment, the mice were habituated to the rotation cages (Rotating Animal Cage System, RACS, AgnTho's, Lidingö, Sweden) for a minimum of 1 h prior to probe insertion (Fig. 1a–c). Fluorinated ethylene propylene tubing (FEP PTFE tubing, ID 0.12 mm, AgnTho's), FEP Tubing Connector Peristaltic Kit (CMA Microdialysis AB, Kista, Sweden), and the probe (CMA7, MWCO 2 MDa 1 mm, CMA microdialysis AB) pre-coated with 5% PEI (MW ∼ 2000, Sigma Aldrich, Saint Louis, MO, USA), were connected to each other and to the push-pull microdialysis set-up as described previously (Fig. 1a) [15]. The set-up contained: a CMA 402 Microdialysis syringe pump (CMA Microdialysis AB), a Reglo ICC Digital Peristaltic pump (CMA Microdialysis AB), and a CMA 470 Refrigerated Microfraction Collector (CMA Microdialysis AB) set to 4 °C, 30 min (during the day) and 60 min (during the night).

The mice were anesthetized with isoflurane, connected to the rotation cage sensor and the probe was inserted into the guide cannula. The probe was perfused with 0.15% BSA (Sigma Aldrich) in Ringer solution (10x Ringer: 147 mM NaCl, 1.2 mM CaCl_2_ x H_2_O, 2.7 mM KCl, 1.0 mM MgCl_2_ x 6 H_2_O) at a flow rate of 0.5 μL/min. The mice were allowed to recover for approximately 20 h to minimize probe-induced tissue damage and prevent sampling of analytes released from injured tissue.

On the following day, 35 ± 4.2 nmol/kg of radiolabeled [^125^I]RmAb158-scFv8D3 (23 ± 4.5 Bq/ng antibody) or 35 ± 2.4 nmol/kg of [^125^I]RmAbSynO2-scFv8D3 (23 ± 5.4 Bq/ng antibody), was injected intravenously to anesthetized mice. This corresponds to an injected dose of 5 mg/kg body weight, expressed as IgG equivalents. Dialysate samples were collected for 24 h after injection. The vials (Polypropylene sample vials 300 μL, AgnTho's) were weighed before and immediately after sample collection to determine the collected volume. If the fluid recovery was lower or higher than 97–103%, the peristaltic pump flow rate was adjusted as described by Chang et al. [24]. The vials collected during the night were weighed the following morning and the sample volume was subsequently corrected for an evaporation constant, which was experimentally determined for each microdialysis experiment by measuring the weight of six vials filled with dialysis buffer before and after overnight evaporation in the same room as the microanalysis was conducted. A constant was then calculated as the mean weight loss per hour and ranged from 0.11 to 0.16 mg/h. Blood samples were continuously collected using 8 μL capillaries from the tail vein at fixed time points after antibody injection (Fig. 1c).

After completion of the microdialysis, the mice were transcardially perfused with saline, and the brain and terminal blood were collected. A subset of mice (n = 6 per antibody group) that did not undergo microdialysis sampling were perfused at 12 h after injection. The brain's hemispheres were divided, and the left hemisphere was kept intact, while the cerebellum was removed from the right hemisphere. Both hemispheres and the cerebellum were stored at −20 °C until further analysis.

### *In vitro* probe recovery

To determine the *in vitro* probe recovery, the probe was placed into an Eppendorf tube filled with a known concentration of [^125^I]RmAb158-scFv8D3 or [^125^I]RmAbSynO2-scFv8D3 in 0.15% BSA in Ringer solution (5.4 μg/mL (123 ± 43 Bq/uL) and 4.9 μg/mL (121 ± 77 Bq/uL) respectively) from the same batch of radiolabeled antibody as used in the *in vivo* microdialysis. The *in vitro* probe recovery was calculated by dividing the dialysate concentration (C_dialysate_) with the concentration in the external medium (C_ext_) for each individual microdialysis probe according to the following formula:

$$In\;vitro\;probe\;recovery=\frac{C_{dialysate}\;}{C_{ext}}$$

The average probe recovery was 2.1 ± 1.1% (mean ± SD). The concentration in the brain ISF (C_ISF_) was calculated by dividing the dialysate concentration with the *in vitro* probe recovery.

$$C_{ISF}=\frac{C_{dialysate}\;}{probe\;recovery}$$

### Brain tissue extraction for *ex vivo* bio-distribution study

Brains from animals euthanized at 12 h after injection (n = 6 per antibody), as well as from a subset of animals that underwent microdialysis (n = 6 injected with [^125^I]RmAb158-scFv8D3 and n = 7 injected with [^125^I]RmAbSynO2-scFv8D3) were homogenized to obtain extracts of different solubility. The cerebrum of the right brain hemisphere, contralateral to the implanted microdialysis probe, was homogenized and sequentially extracted with a 1:5 wt/volume ratio with 1) Tris-buffered saline (TBS), 2) TBS with 0.5% Triton X-100 (TBST) and 3) 70% formic acid (FA) to release soluble Aβ (monomers, oligomers and protofibrils), membrane-associated Aβ (monomers, oligomers and protofibrils) and total plaque-associated Aβ (monomerized), respectively (Fig. 1d). Briefly, the tissue was homogenized using a Precellys Evolution system (Bertin Technologies SAS, Montigny-le-Bretonneux, France). The TBS homogenate was centrifuged for 1 h at 16 000×*g* before the supernatant (TBS_16K_) was collected and divided into two separate tubes, one of which was further centrifuged for 1 h at 100 000×*g* using a Himac Tabletop Mikro Ultracentrifuge CS150NX (Hitatchi, Tokyo, Japan) followed by collection of the supernatant (TBS_100K_). The TBS pellet was re-homogenized with 0.5% TBST and centrifuged at 16 000×*g* for 1 h. The homogenization and centrifugation steps were repeated with FA (Fig. 1d). The radioactivity of all homogenate samples was measured with a γ-counter (2480 Wizard™, Wallac Oy PerkinElmer, Turku, Finland), and the samples were stored at −20 °C for further analysis by ELISA. The radioactivity concentration was quantified as the fraction of injected dose per gram tissue, corrected for the body weight of the animal (ID/g/bw).

$$ID/g/bw=\frac{measured\;radioactivity\;per\;gram\;tissue}{total\;injected\;radioactivity\;per\;gram\;body\;weight}$$

$$Brain\;to\;blood\;ratio=\frac{radioactivity\;per\;gram\;brain}{radioactivity\;per\;gram\;blood}$$

### Aβ, TREM2 and cytokine ELISAs

To measure the concentration of Aβ aggregates, Aβ1-40, Aβ1-42 and soluble triggering receptor expressed on myeloid cells 2 (TREM2), different sandwich ELISAs were used. Soluble Aβ aggregates were measured with a sandwich ELISA using the N-terminal-specific antibody 3D6 (produced in house) both as a capture and detection antibody. This allows detection of aggregates from a dimer and larger, while excluding monomeric Aβ [25]. Soluble Aβ aggregates were measured in TBS_100K_ and TBS_16K_ brain extracts. Membrane-associated or intracellular Aβ aggregates were measured in TBST brain extracts.

COSTAR 96-well half-area plates were coated with 50 ng of 3D6 antibody per well and incubated at 4 °C overnight. The plates were blocked for 2 h with 1% BSA before brain extracts and a protofibril calibration standard were added and incubated overnight at 4 °C. The calibration standard was prepared by size exclusion chromatography purification of aggregated Aβ1-42 (Innovagen, Lund, Sweden) [25]. Biotinylated 3D6, 25 ng, was added and incubated for 2 h at RT. Streptavidin-HRP (1:2000, Mabtech AB, Nacka, Sweden) was added and incubated for 1 h followed by development with K-blue Aqueous TMB substrate (Neogen Corp., Lexington, USA). The plate was read with a Tecan Infinite M200 PRO spectrophotometer at 450 nm and the samples were quantified against the standard curve.

Total Aβ1-40 and Aβ1-42 were analyzed in FA brain extracts. Briefly, 96-well half-area plates were coated overnight at 4 °C with 50 ng anti-Aβ40 (custom production, Agrisera, Umeå, Sweden) or anti-Aβ42 (ThermoScientific, Waltham, MA, US) antibodies. The plates were blocked with 1% BSA for 2 h at RT. FA samples were neutralized with 2 M tris buffer and added to the plate. Aβ1-40 (Innovagen, SP-BA40-1) or Aβ1-42 (Innovagen, SP-BA42-1) standards were also loaded onto the plate and incubated overnight at 4 °C. The following day, the samples were detected with biotinylated 3D6, streptavidin-HRP and developed using K-blue Aqueous TMB substrate as described above.

The concentrations of soluble TREM2 in TBS_16K_ and TBS_100K_ brain extracts were analyzed with ELISA as previously described [26]. Briefly, 96-well plates were coated with 25 ng per well of AF1729 (R&D Systems) and incubated overnight at 4 °C. The plates were then blocked with 1% BSA for 2 h at RT. The brain extracts were loaded onto the plate together with the TREM2 standard (Sino Biological, 158–50149-M02H-100) and incubated overnight at 4 °C. The biotinylated detection antibody BAF1729 (R&D Systems) was loaded and incubated for 2 h at RT, followed by streptavidin-HRP and development with K-blue Aqueous TMB substrate as described above.

The pro-inflammatory cytokines IL-1β, IL-6, and TNF-α were measured in TBS_16K_ brain extracts using ELISA Flex kits (Mabtech AB), according to the manufacturer's instructions.

### Statistics

Data are presented as mean ± standard deviation (SD). Unpaired *t*-test, Two-way ANOVA followed by Tukey's multiple comparisons test and Mixed effects model followed by Šídák's multiple comparisons test were performed. Correlations were analyzed using Pearson correlation test (r) and simple linear regression (R^2^). All tests were two-tailed, with a significance level set at 5% (α = 0.05). Statistically significant differences were defined as follows: p-value <0.05 (∗), p-value <0.01 (∗∗), p-value <0.001 (∗∗∗), p-value <0.0001 (∗∗∗∗). Graphs and statistical analyses were performed using GraphPad Prism version 10.6.1 (GraphPad Software, San Diego, California, USA).

## Results

Antibody blood and ISF pharmacokinetics and antibody distribution in brain tissue.

**Fig. 2:**
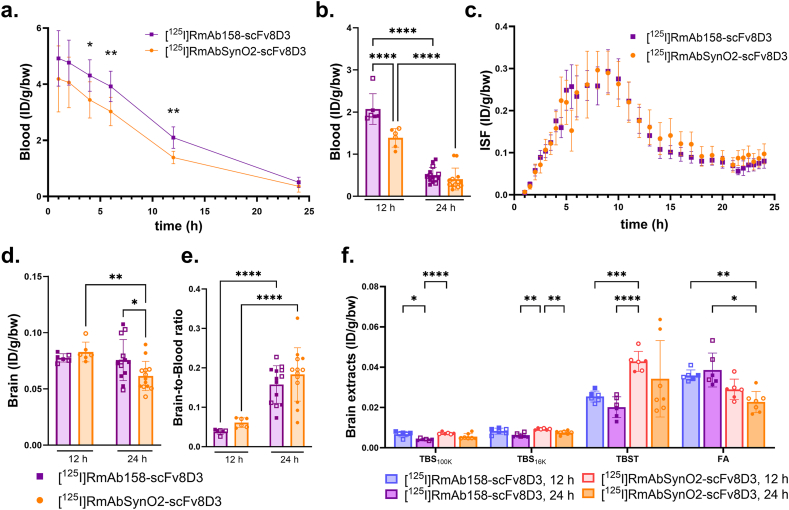
a. Blood concentrations of [^125^I]RmAbSynO2-scFv8D3 and [^125^I]RmAbSynO2-scFv8D3, 1–24 h p.i. in tg-ArcSwe mice, expressed as the fraction of the injected dose per gram tissue, corrected for body weight (ID/g/bw). b. Blood concentrations (ID/g/bw) at 12 h and 24 h p.i. c. Brain ISF concentrations (ID/g/bw), 1–24 h p.i. d. Brain concentrations (ID/g/bw) at 12 h and 24 h p.i. e. Brain to blood ratio at 12 h and 24 h p.i. f. Antibody concentrations (ID/g/bw) in TBS_100K_, TBS_16K_, TBST and FA brain homogenate fractions at 12 h and 24 h p.i. Animal numbers: 12 h p.i. (n = 6 per group); 24 h p.i. (n = 13 per group). Filled symbols represent males and open symbols represent females. Mixed effects model followed by Šídák's multiple comparisons test was performed in a and c. Two-way ANOVA followed by Tukey's multiple comparisons test was performed in b and d-f. (∗p < 0.05, ∗∗p < 0.01, ∗∗∗p < 0.001, ∗∗∗∗p < 0.0001). All data is presented as mean ± SD. Statistical analyses and corresponding results, including details on the specific tests applied to each dataset, are provided in Table S1.

Blood concentrations of the antibodies [^125^I]RmAb158-scFv8D3 and [^125^I]RmAbSynO2-scFv8D3 were measured in blood over 24 h p.i. in tg-ArcSwe mice. [^125^I]RmAb158-scFv8D3 exhibited a slower blood clearance between 4 and 12 h p.i., before reaching the same level as [^125^I]RmAbSynO2-scFv8D3 at 24 h p.i (Fig. 2a and b). Concentrations of freely diffusible antibody in the brain (i.e. the brain ISF concentration) were determined by measuring the radioactivity in microdialysates collected 1–24 h post injection (p.i.) (Fig. 1b). The ISF concentrations of [^125^I]RmAb158-scFv8D3 and [^125^I]RmAbSynO2-scFv8D3 were similar throughout the 24 h measurement period, with the highest concentrations (C_max_) measured at 9 and 8 h p.i., respectively (Fig. 2c). Notably, at 24 h p.i., the mean concentration of both antibodies was already reduced to about 25% of C_max_. No difference in brain concentrations was detected between the antibodies at 12 h p.i, whereas 24 h p.i. There were significantly higher brain concentrations of [^125^I]RmAb158-scFv8D3 in comparison to [^125^I]RmAbSynO2-scFv8D3 (Fig. 2d). However, when the brain concentrations were normalized to the blood concentrations, this difference between the antibodies disappeared (Fig. 2e).

To determine how [^125^I]RmAb158-scFv8D3 interacts with Aβ pools of different solubility, brain tissue homogenates were sequentially extracted according to increasing solubility (Fig. 1d). At both 12 h and 24 h p.i., [^125^I]RmAb158-scFv8D3 and [^125^I]RmAbSynO2-scFv8D3 were primarily detected in the less soluble fractions (TBST and FA), with [^125^I]RmAb158-scFv8D3 reaching the highest concentration in the FA fraction and [^125^I]RmAbSynO2-scFv8D3 in the TBST fraction (Fig. 2f). The [^125^I]RmAb158-scFv8D3 concentration tended to decrease over time in all fractions except the FA fraction, indicating a time-dependent redistribution of the antibody from the more soluble fractions toward the insoluble, plaque-associated FA fraction. In contrast, the [^125^I]RmAbSynO2-scFv8D3 concentrations tended to decrease with time in all brain fractions, suggesting clearance or degradation of the antibody.

### Antibody effects on Aβ concentrations in the brain

**Fig. 3:**
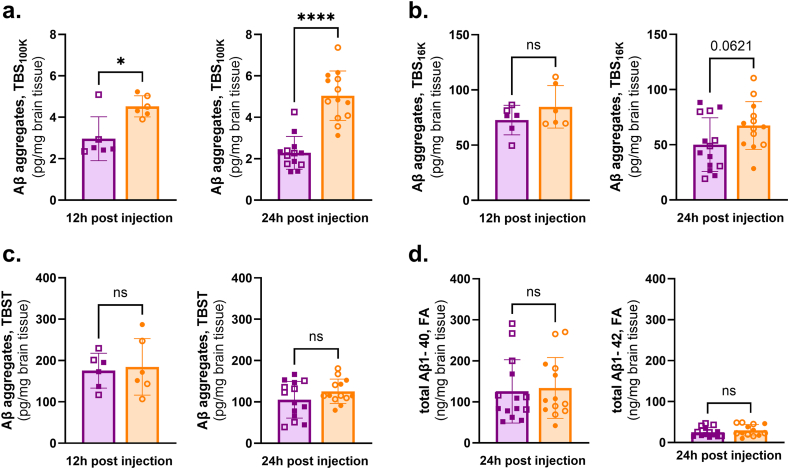
Aβ concentrations in different brain homogenate fractions after antibody treatment. a. Soluble Aβ aggregates in the TBS_100K_ fraction at 12 h and 24 h p.i. b. Soluble Aβ aggregates in the TBS_16K_ fraction at 12 h and 24 h p.i. c. Soluble Aβ aggregates in the TBST fraction at 12 h and 24 h p.i. d. Total Aβ1-40 and Aβ1-42 in the FA fraction at 24 h p.i. Filled symbols represent males and open symbols represent females. Unpaired *t*-test (∗p < 0.05, ∗∗∗∗p < 0.0001), mean ± SD. Statistical analyses and corresponding results, including details on the specific tests applied to each dataset, are provided in Table S1.

To investigate whether [^125^I]RmAb158-scFv8D3 had a therapeutic effect on brain Aβ concentrations, soluble Aβ aggregates as well as total Aβ1-40 and Aβ1-42, were measured with ELISA in the different brain homogenate fractions. The [^125^I]RmAb158-scFv8D3 treated group displayed lower concentrations of the most soluble Aβ aggregates, i.e. those present in the TBS_100K_ fraction, at both 12 h and 24 h p.i., in comparison to the [^125^I]RmAbSynO2-scFv8D3 treated group, with a more pronounced effect at 24 h p.i. (Fig. 3a). No difference in concentrations of Aβ aggregates was observed between the groups in the TBS_16K_ or TBST fractions (Fig. 3b and c). Still, a trend toward lower concentrations of Aβ aggregates in TBS_16K_ for the [^125^I]RmAb158-scFv8D3 treated group could be observed 24 h p.i. (Fig. 3b). Aβ aggregates cannot be measured in brain homogenates extracted with FA, which will disintegrate aggregates. Thus, total levels of monomerized Aβ were measured in the FA brain homogenate fractions. There were no differences in either Aβ1-40 or Aβ1-42 concentrations between the [^125^I]RmAb158-scFv8D3 and [^125^I]RmAbSynO2-scFv8D3 treated groups at either of the time points (Fig. 3d, Fig. S1). Soluble Aβ aggregates as well as total Aβ1-40 and Aβ1-42 were below the detection limit in the microdialysates.

### Antibody effects on TREM2

**Fig. 4:**
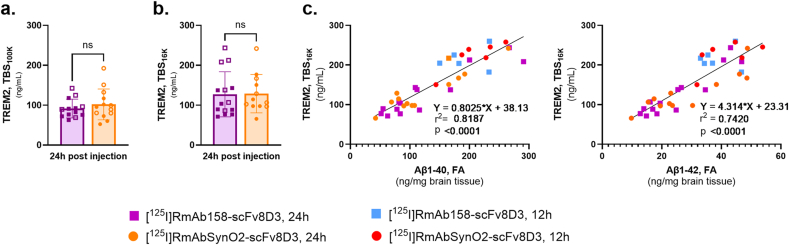
a. Soluble TREM2 in TBS_100K_ 24 h p.i. b. Soluble TREM2 in TBS_16K_ 24 h p.i. Filled symbols represent males and open symbols represent females. Unpaired *t*-test, mean ± SD. c. Correlation and simple linear regression between TREM2 in TBS_16K_ and total Aβ1-40 and Aβ1-42 in FA (Pearson r). Statistical analyses and corresponding results, including details on the specific tests applied to each dataset, are provided in Table S1.

The microglial protein TREM2 was measured in the TBS_100K_ and TBS_16K_ brain homogenate extracts from mice treated with [^125^I]RmAb158-scFv8D3 or [^125^I]RmAbSynO2-scFv8D3. No difference in TREM2 levels in the TBS_100K_ or TBS_16K_ extracts could be observed between the two antibody treatments at either 12 h (Fig. S2) or 24 h p.i. (Fig. 4a and b). Despite this, there was a strong correlation between total plaque-associated Aβ and TREM2 levels, as illustrated by the relationship between TREM2 and Aβ1-40 as well as TREM2 and Aβ1-42 in the FA fraction (Fig. 4c).

### Antibody effects on proinflammatory cytokines

**Fig. 5:**
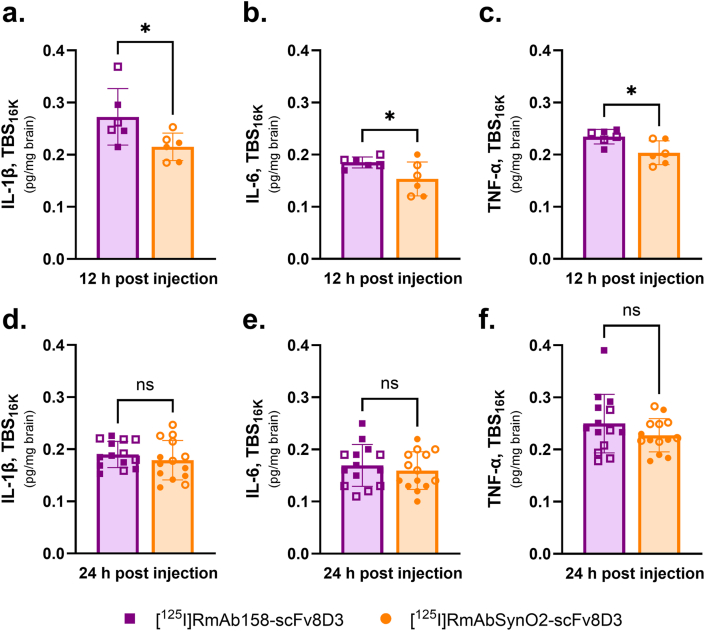
ELISA quantification of the proinflammatory cytokines IL-1β, IL-6, and TNF-α in TBS_16K_ brain extracts obtained 12 h p.i. (a-c) and 24 h p.i. (d-f). Filled symbols represent males and open symbols represent females. Unpaired *t*-test (∗p < 0.05), mean ± SD. Statistical analyses and corresponding results, including details on the specific tests applied to each dataset, are provided in Table S1.

The proinflammatory cytokines IL-1β, IL-6 and TNF-α were measured in the TBS_16K_ brain extracts from mice treated with [^125^I]RmAb158-scFv8D3 or [^125^I]RmAbSynO2-scFv8D3. At 12 h after injection all three cytokines were elevated in the brains of mice that received [^125^I]RmAb158-scFv8D3 compared to [^125^I]RmAbSynO2-scFv8D3 (Fig. 5a–c). At 24 h after antibody injection, the elevation in cytokine release was resolved and no difference was seen between the groups (Fig. 5 d-f).

## Discussion

The present study examined the distribution of the bispecific antibody RmAb158-scFv8D3 during the first 24 h after administration, from blood into the brain interstitial fluid (ISF) and further into the parenchyma in the tg-ArcSwe mouse model of Aβ pathology. By comparing the dynamics of RmAb158-scFv8D3 distribution to the α-synuclein-directed antibody RmAbSynO2-scFv8D3, which lacks a primary brain target, we were able to dissect the earliest steps of antibody action in the brain. This approach provides insight into how a bispecific therapeutic antibody traffics within the brain, initiates interactions with Aβ, and therapeutically affects distinct pools of aggregated Aβ. TfR-mediated antibody transport across the BBB is a continuous process that gradually declines over time after administration, with clearance of antibody from the blood. In parallel with brain entry, the antibody is cleared from the ISF, either via regular drainage pathways or through protein interactions that promote redistribution from the ISF into the brain parenchyma. RmAb158-scFv8D3 is expected to primarily interact with various forms of Aβ in the brain, which are likely to dominate its intraparenchymal distribution and retention, although TfR-mediated interactions may also contribute. For RmAbSynO2-scFv8D3, in contrast, interactions with TfR expressed on cells such as neurons and astrocytes [12] may be more prominent and could lead to cellular uptake, endocytosis, and antibody degradation.

After i.v. administration, [^125^I]RmAb158-scFv8D3 and [^125^I]RmAbSynO2-scFv8D3 exhibited comparable concentration trajectories in brain ISF over 24 h, with C_max_ at 8–9 h p.i. and most of both antibodies cleared from ISF by the end of the measurement period. These results extend findings from a previous study where we measured ISF concentrations of a bispecific antibody at shorter time intervals, 1–6 h and 21–24 h after injection, using the same technique [15]. Notably, at 12 h p.i., ISF concentrations of both antibodies were about 2.5-fold higher than the total brain concentrations. Taking into consideration that the volume of ISF is about 20% of the brain's total volume [27], this suggests that about half of the injected antibody resided in ISF at 12 h p.i., and then declined to about 25% at the end of the experiment, indicating a global relative redistribution from ISF to parenchyma over time. At 24 h p.i. There was more [^125^I]RmAb158-scFv8D3 retained in the brain tissue than [^125^I]RmAbSynO2-scFv8D3, demonstrating substantial binding to Aβ in the brain. Even though the affinity of RmAb158-scFv8D3 is reportedly 10 times higher toward soluble Aβ protofibrils than fibrillar Aβ *in vitro*, the majority of the [^125^I]RmAb158-scFv8D3 target binding was detected in the FA extracted homogenate containing insoluble Aβ. This is in accordance with lecanemab reducing the amyloid PET signal (representing Aβ plaques) in AD patients [6]. Moreover, it suggests that [^125^I]RmAb158-scFv8D3 is specifically redistributed from a free unbound state in the ISF to a target-bound state in the plaque-containing FA-fraction. Here, we were unable to detect soluble Aβ aggregates in the ISF, indicating that their concentrations are very low. Consequently, most of the antibody is likely to encounter its target Aβ in plaques, where Aβ is predominantly present in its fibrillar form, despite the antibody's somewhat lower affinity for fibrillar Aβ. Indeed, previous work has shown that a considerable amount of antibody remains bound to plaques even after four weeks [13]. *In vitro* work has demonstrated how lecanemab targets diffusible fibrils which share cryo-EM structures with plaque-derived fibrils [28]. However, the proportion of soluble versus plaque-associated Aβ targeted by the antibody will likely be dependent on disease stage, favoring plaque-associated Aβ at later disease stages.

While higher concentrations of [^125^I]RmAb158-scFv8D3 were measured in the FA-fraction, an immediate Aβ-reducing effect of the antibody was observed in the TBS_100K_ fraction already at 12 h p.i, with an even stronger effect at 24 h p.i. The sequential homogenization of brain tissue collected the smallest, most soluble brain tissue content in the TBS_100K_ fraction, likely capturing also aqueous Aβ fibrils which share cryo-EM structures with plaque-derived fibrils [28]. Still, it is important to note that this pool of Aβ only accounts for a small portion of the total Aβ load in both AD patients [29] and in the tg-ArcSwe mice, where the TBS_100K_ fraction contains >10 000-fold less Aβ than the FA fraction. This Aβ pool is therefore more sensitive to acute therapeutic intervention [5,11,30]. In previous work, repeated injections of RmAb158-scFv8D3 have been able to markedly reduce the levels of FA-soluble Aβ1-42, located at the border of insoluble Aβ plaques in *App*^*NL-G-F*^ and tg-ArcSwe mice [31,32]. The single injection used in this current study did not alter the FA-soluble Aβ1-42 levels. With a longer treatment regimen, we would expect to see larger changes in FA-soluble Aβ1-42 rather than Aβ1-40, given the more accessible location of Aβ1-42 within the ‘halo’ of the plaques. In addition, Aβ1-40 deposition in tg-ArcSwe mice is highly abundant in vascular deposits, as in the human AD brain, which are less targeted by bispecific antibodies that rely on TfR-mediated transport across the brain's network of capillaries, thus avoiding the perivascular space of large amyloid-laden brain vessels [9].

Accumulating evidence suggests that the main clearance mechanism in Aβ-directed antibody therapy is mediated by microglia. Through their Fc-gamma receptors, these cells interact with the Fc domain of antibodies bound to Aβ plaques in the brain, inducing phagocytosis and degradation of antibody–Aβ complexes [33]. This process has been suggested to affect the expression of TREM2, although it is not fully elucidated whether changes in brain TREM2 levels following antibody therapy occur as a direct response to the treatment itself or as a consequence of treatment-induced reduction in Aβ pathology [34]. In line with previous studies, soluble TREM2 levels in the brain showed a strong positive correlation with plaque-associated Aβ [20,26,35,36]. However, during the short timeframe of this study, the subtle changes in overall Aβ levels induced by the antibody treatment did not elicit any changes in TREM2 levels. While TREM2 upregulation may be a relatively slow process, immune cells, including microglia, can respond rapidly through cytokine secretion. Anti-Aβ antibody therapy has been reported to elicit a pro-inflammatory cytokine response [37]. In the present study, a small but consistent increase in the pro-inflammatory cytokines IL-1β, IL-6, and TNF-α was observed at 12 h p.i. in brain extracts from mice treated with [^125^I]RmAb158-scFv8D3 compared with [^125^I]RmAbSynO2-scFv8D3. Interestingly, at 24 h p.i., when most of the antibody had been cleared from the ISF, cytokine levels returned to baseline. This suggests a subtle pro-inflammatory microglial response to antibody treatment that correlates with ISF concentration of the antibody and is rapidly resolved upon antibody clearance.

Microdialysis provides the opportunity to measure antibody concentrations at their site of action in the brain and enables different compartments, such as ISF and cerebrospinal fluid, to be studied in a living model. One challenge when performing microdialysis with large molecules, such as antibodies, is the risk of ultrafiltration of perfusion fluid out of the probe when using membranes with large pore sizes [15,24,38,39]. This may dilute the dialysates, which then require more sensitive analysis techniques. In this current study, Aβ aggregates could not be measured in the microdialysates. We have previously reported a size range of 80–500 kDa for mAb158-detected Aβ aggregates in both human and mouse brain tissue [3]. This is well below the molecular weight cutoff of the microdialysis probe, suggesting that low concentration rather than too large size limited the detection of Aβ aggregates. A limitation of the current study is that probe recovery was determined only *in vitro* and not *in vivo* using retrodialysis. Another limitation is that microdialysis was performed in the hippocampus, whereas whole cerebrum samples were used to assess global brain concentrations of the antibody, as well as Aβ, TREM2, and cytokines.

In summary, a single injection of the bispecific RmAb158-scFv8D3 antibody thus constitutes a useful model of how the antibody distributes in the brain and takes part in plaque clearance. This study revealed a short antibody residence time in the ISF, with the majority eliminated or redistributed to the plaque containing brain fraction within 24 h p.i. In combination with an immediate reduction in soluble Aβ aggregates, this suggests that continuous dosing of the antibody would result in a rapid voyage through the ISF, where a small portion of the injected antibody would bind and neutralize the scarce soluble Aβ aggregates, while the rest would target the abundant insoluble Aβ aggregates that constitute the plaques. Once the antibody is in a plaque-bound state, there is ample time for microglia to phagocytose and degrade the antibody-decorated plaque. Newly arriving antibody continues the plaque-decoration and thereby keeps recruiting microglia to clear the plaque remnants.

## Author contributions

EW: Writing – original draft, Methodology, Formal analysis, Data curation, Conceptualization; AD: Writing – original draft, Methodology, Formal analysis, Data curation; UJ: Writing – review & editing, Methodology, Conceptualization; MX: Writing – review & editing, Methodology; WM: Writing – review & editing, Data curation, Conceptualization; SS: Writing – review & editing, Funding acquisition, Formal analysis, Data curation, Conceptualization; DS: Writing – review & editing, Methodology, Funding acquisition, Formal analysis, Data curation, Conceptualization.

## Data availability

The datasets used and/or analyzed during the current study are available from the corresponding author on reasonable request.

## Funding

This study was funded by the 10.13039/501100004359Swedish Research Council (2021-01083, 2021-03524, 2024-02963 and 2025-02940), 10.13039/501100008599Alzheimerfonden, Hjärnfonden, Åhlén-stiftelsen, Konung Gustaf V:s och Drottning Victorias frimurarestiftelse, Stohnes stiftelse, and Stiftelsen för Gamla Tjänarinnor. The funding organizations did not take part in designing the study, in collecting, analyzing, or interpreting the data, or in writing the manuscript.

## Declaration of competing interests

The authors declare the following financial interests/personal relationships which may be considered as potential competing interests:

Dag Sehlin reports financial support and article publishing charges were provided by Uppsala University. If there are other authors, they declare that they have no known competing financial interests or personal relationships that could have appeared to influence the work reported in this paper.
