# Supplementary material for: Brain interstitial fluid pharmacokinetics and therapeutic effect of a BBB penetrating amyloid beta antibody measured by microdialysis
Source: Neurotherapeutics. 2026 Jun 24;23(4):e00949. doi: 10.1016/j.neurot.2026.e00949 (PMC13319731; doi:10.1016/j.neurot.2026.e00949)
Supplement: Multimedia component 1 [file mmc1.docx]

Brain interstitial fluid pharmacokinetics and therapeutic effect of a BBB penetrating amyloid beta antibody measured by microdialysis

Elin Wik*, Amelia D Dahlén*, Ulrika Julku*, Mengfei Xiong*†, Wojciech Michno*, Stina Syvänen* and Dag Sehlin*

^*^Department of Public Health and Caring Sciences, Section of Molecular Geriatrics, Uppsala University, Sweden

^†^*Department of Department of Medicinal Chemistry, Uppsala University, Uppsala, Sweden*

Correspondence: [dag.sehlin@uu.se](mailto:dag.sehlin@uu.se)

Supplementary information:

| **Table S1.** Statistical analyses | | | | |
| --- | --- | --- | --- | --- |
| **Figure** | **Test** | **Row factor: Time**  **F (DFn, DFd)** | **Column factor:**  **Treatment**  **F (DFn, DFd)** | **Time x Treatment**  **F (DFn, DFd)** |
| Fig. 2a | Mixed-effects model (REML) | F (2.572, 29.54) = 22.80  p<0.0001 | F (1, 14) = 0.04753  p = 0.8306 | F (2.572, 29.54) = 0.5124  p = 0.6495 |
| Fig. 2b | Mixed-effects model (REML) | F (1.686, 24.95) = 218.6  p<0.0001 | F (1, 36) = 14.63  p=0.0005 | F (1.686, 24.95) = 1.658  p=0.2127 |
| Fig. 2c | Two-way ANOVA | F (1, 39) = 221.0  P<0.0001 | F (1, 39) = 21.59  P<0.0001 | F (1, 39) = 12.57  P=0.001 |
| Fig. 2d | Two-way ANOVA | F (1, 34) = 5.853  P=0.0210 | F (1, 34) = 0.8478  P=0.3637 | F (1, 34) = 4.064  P=0.0518 |
| Fig. 2e | Two-way ANOVA | F (1, 34) = 49.07  P<0.0001 | F (1, 34) = 1.949  P=0.1717 | F (1, 34) = 0.005770  P=0.9399 |
| Fig. 2f | Two-way ANOVA | F (1.391, 29.22) = 130.9  P<0.0001 | F (3, 21) = 3.070  P=0.0501 | F (4.174, 29.22) = 7.456  P=0.0002 |
| **Figure** | **Test** | **P value** |  |  |
| Fig. 3a  12 h p.i. | Unpaired t test | 0.0135 |  |  |
| Fig. 3a  24 h p.i. | Unpaired t test | <0.0001 |  |  |
| Fig. 3b  12 h p.i. | Unpaired t test | 0.2379 |  |  |
| Fig. 3b  24 h p.i. | Unpaired t test | 0.0621 |  |  |
| Fig. 3c  12 h p.i. | Unpaired t test | 0.7865 |  |  |
| Fig. 3c  24 h p.i. | Unpaired t test | 0.1749 |  |  |
| Fig. 3d  Total Aβ1-40, FA | Unpaired t test | 0.7787 |  |  |
| Fig. 3d  Total Aβ1-42, FA | Unpaired t test | 0.3593 |  |  |
| **Figure** | **Test** | **P value** |  |  |
| Fig. 4a  TREM2, TBS_100K_ | Unpaired t test | 0.4268 |  |  |
| Fig. 4a  TREM2, TBS_16K_ | Unpaired t test | 0.9381 |  |  |
| Fig. 4c  Aβ1-40, FA | Pearson r | 0.9048 |  |  |
| Fig. 4c  Aβ1-42, FA | Pearson r | 0.8614 |  |  |
| **Figure** | **Test** | **P value** |  |  |
| Fig. 5a  IL-1β, 12 h p.i. | Unpaired t test | 0.0409 |  |  |
| Fig. 5b  IL-6, 12 h p.i. | Unpaired t test | 0.0473 |  |  |
| Fig. 5c  TNF-α, 12 h p.i. | Unpaired t test | 0.0183 |  |  |
| Fig. 5a  IL-1β, 12 h p.i. | Unpaired t test | 0.3747 |  |  |
| Fig. 5e  IL-6, 24 h p.i. | Unpaired t test | 0.4938 |  |  |
| Fig. 5f  TNF-α, 24 h p.i. | Unpaired t test | 0.2034 |  |  |


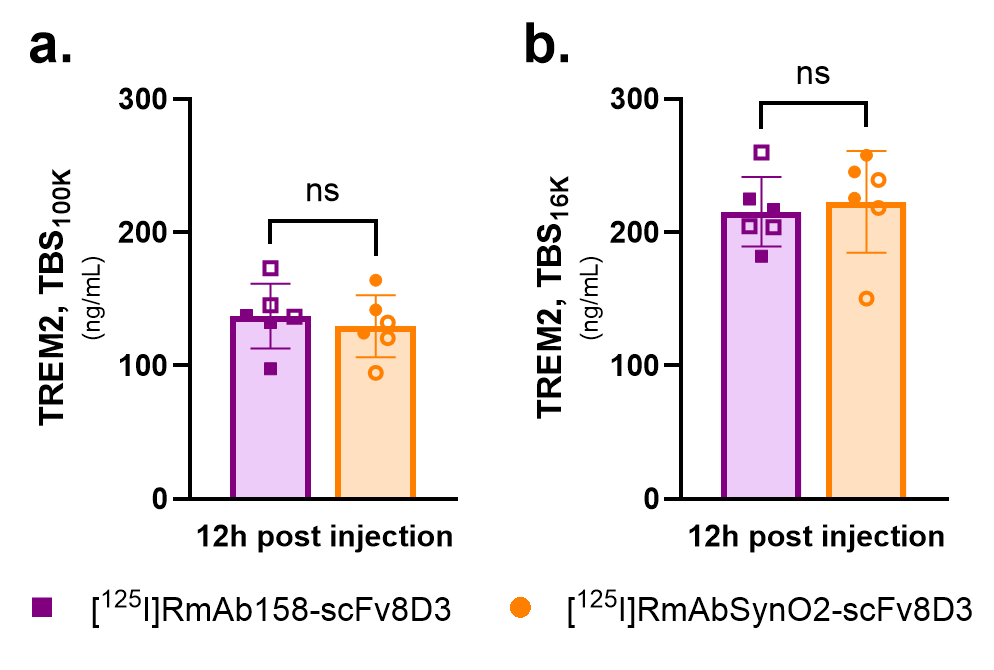

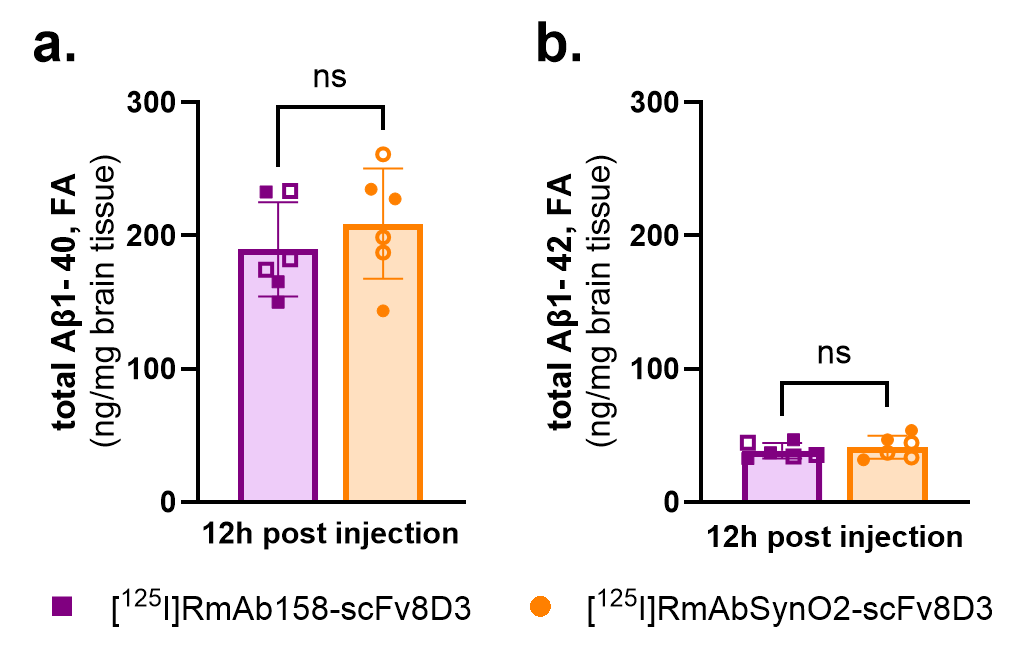
**Fig. S1** Effects of antibody treatment on different homogenate pools of Aβ. **a**. Total Aβ1-40 in formic acid (FA) extracted homogenate at 12 h post injection (p.i.) and **b.** total Aβ1-42 FA extracted homogenate at 12 h p.i. Filled symbols represent males and open symbols represent females. Unpaired t test, mean ± SD

***Fig. S2* a.** TREM2 in TBS_100K_ extracted homogenate at 12 h post injection (p.i.) and **b**. TREM2 in TBS_16K_ extracted homogenate at 12 h p.i. Filled symbols represent males and open symbols represent females. Unpaired t test, mean ± SD
